# Effect of chewing gum on clinical outcomes and postoperative recovery in adult patients after gastrointestinal surgery: an umbrella review

**DOI:** 10.1097/JS9.0000000000002332

**Published:** 2025-03-12

**Authors:** Shu Ling Lew, Ling Jie Cheng, Siat Yee Yap, Yi Qi Liaw, Jiyoung Park, Siew Tiang Lau

**Affiliations:** a Alice Lee Centre for Nursing Studies, Yong Loo Lin School of Medicine, National University of Singapore, Singapore; b Department of Nursing, Ng Teng Fong General Hospital, Singapore, Singapore; c National Perinatal Epidemiology Unit, Nuffield Department of Population Health, University of Oxford, Oxford, United Kingdom; d Saw Swee Hock School of Public Health, National University of Singapore, Singapore; e University Surgical Cluster, National University Hospital, Singapore; f College of Nursing, Institute for Health Science Research, Inje University, Busan, South Korea; g YEIRIN Social Cooperative, Busan, South Korea

**Keywords:** chewing gum, gastrointestinal surgery, postoperative outcomes, postoperative recovery, umbrella review

## Abstract

**Background:**

Gastrointestinal surgery is crucial for many medical conditions but can lead to difficult recoveries. Chewing gum is proposed as a remedy, yet existing reviews offer conflicting results. This umbrella review aims to synthesize the effectiveness of chewing gum on time to first flatus, time to first bowel movement, length of stay and complication rates in adult patients.

**Methods:**

We conducted an umbrella review, searching seven databases up to 17 November 2023, with an updated search extending to 1 January 2025. The focus was on post-surgery chewing gum interventions. The quality and certainty of evidence were assessed using the AMSTAR-2 tool and umbrella review criteria.

**Results:**

Seventeen reviews, encompassing 26 672 participants from 264 primary studies, were included. Meta-analyses indicated reductions in time to first flatus by −0.36 days (95% CI = −0.61, −0.1) or −12.26 hours (95% CI = −14.73, −9.78), time to first bowel movement by −0.59 days (95% CI = −0.94, −0.23) or −19.29 hours (95% CI = −23.79, −14.79), and length of stay by −0.85 days (95% CI = −1.22, −0.48) or −20.08 hours (95% CI = −28.62, −11.54). Additionally, chewing gum was associated with fewer postoperative complications.

**Conclusion:**

Chewing gum may significantly aid postoperative care by reducing time to first flatus, time to first bowel movement, and length of stay. However, many included reviews were of low quality with weak evidence, highlighting the need for more rigorous studies to confirm these benefits. Integrating chewing gum into clinical practice could enhance recovery and optimize hospital bed turnover, making it a valuable addition to postoperative care protocols.

**HIGHLIGHTS**

- Chewing gum may aid postoperative care, especially for open surgery, non-cancer patients, by reducing time to first flatus, bowel movement, and length of stay.
- Many included reviews were of critically low quality with weak evidence.
- There is a need for more rigorous methodologies in future reviews to confirm these benefits.

## Introduction

Gastrointestinal surgery, including appendectomy, colectomy, and gastrectomy, is among the most common surgical procedures. Appendicitis, a frequent emergency, affects approximately 233 per 100 000 people annually^[^1^]^, while colorectal cancer, the third most common cancer globally, is projected to account for 3.2 million new cases annually by 2040^[^2^]^. Recovery from gastrointestinal surgery is complex, impacting quality of life and clinical outcomes. Postoperative issues like delayed flatus and bowel movement can lead to complications, including postoperative ileus (POI), nausea, vomiting, and prolonged hospital stays, increasing healthcare costs. Despite various strategies to improve postoperative recovery, such as epidural analgesia, minimally invasive surgery, and early mobilization or feeding, systematic reviews show mixed evidence on reducing these complications^[^3-9^]^.

Epidural analgesia reduces time to first flatus (TFF) and bowel movement (TFBM), decreases postoperative pain, and shortens hospital stays for open surgery^[^10^]^. Laparoscopic resection for colorectal cancer offers similar benefits^[^11^]^. Early ambulation aids recovery but has limited impact on hospital stays^[^12^]^. Early feeding enhances gastrointestinal function, though its effects on length of stay remain inconsistent^[^13-15^]^. Studies report reductions in hospital stays with early enteral feeding, yet variations in effectiveness and high costs raise concerns about feasibility and cost-effectiveness. Given the high prevalence of postoperative complications, exploring more sustainable and cost-effective approaches is crucial.

Chewing gum might aid post-gastrointestinal surgery by stimulating the cephalic phase of digestion, which begins with the sight, smell, taste, and chewing of food^[^16^]^. Chewing gum sends signals to the brain that food is being consumed^[^17^]^, triggering saliva release, activating the cephalic phase of digestion and digestive enzyme secretion^[^17^]^. This may help accelerate recovery by promoting gastrointestinal function before actual food intake. However, evidence on its impact on time to first flatus (TFF), bowel movement (TFBM), and length of stay (LOS) is inconclusive^[^3-8,18-28^]^ due to heterogeneity in patient demographics, surgical procedures, and perioperative care^[^19-23^]^.

Given these inconsistencies, this umbrella review will synthesize evidence on chewing gum’s effects on TFF, TFBM, and LOS in adults undergoing gastrointestinal surgery. While recent review suggested gum chewing may reduce postoperative ileus, findings were based on limited studies, potentially overestimating effects^[^29^]^. Therefore, this umbrella review aims to examine chewing gum’s effects on TFF, TFBM, and LOS in adult patients undergoing gastrointestinal surgery. In addition, the review aims to evaluate the effectiveness of chewing gum across various surgical procedures, cancer statuses, chewing frequencies, and durations, and assess the prevalence of complications.

## Methods

### Reporting guideline and protocol registration

This umbrella review adhered to the Joanna Briggs Institute methodology for umbrella reviews^[^30^]^ and the Preferred Reporting Items for Overviews of Systematic Reviews (PRIO-harms) reporting standards^[^31^]^ (Supplementary Digital Content, Table A1, http://links.lww.com/JS9/D994). This review has also been reported in line with Preferred Reporting Items for Systematic Reviews and Meta-Analyses (PRISMA 2020)^[^32^]^ and Assessing the methodological quality of systematic reviews (AMSTAR-2)^[^33^]^ guidelines. The study protocol was registered in International Prospective Register of Systematic Reviews (CRD42024519952).

### Eligibility criteria

Adult patients (>18 years old) who underwent gastrointestinal surgery, regardless of gender and ethnicity were included in this review. Studies on obstetrics and gynecology, urology and pediatrics were excluded to allow for a more in-depth analysis of the effects of chewing gum specifically in this context, without the potential confounding effects of other medical conditions or surgeries. This review focused on chewing gum as intervention, comparing it with standard postoperative care or alternative intervention. Reviews investigating primary outcomes such as TFF, TFBM, and LOS, as well as secondary outcomes concerning the prevalence of postoperative complications were included. Only systematic reviews and meta-analyses in peer-reviewed journals were considered, and only full-text articles in English will be included, with no restrictions on publication dates. The detailed eligibility criteria are presented in Supplementary Digital Content, Table A2, http://links.lww.com/JS9/D994.

### Information sources

A comprehensive three-step search strategy was employed to identify relevant reviews published in English. Initially, a search was conducted in PROSPERO to check for similar umbrella reviews and prevent duplication. This was followed by systematic searches in six databases: CINAHL (EBSCO), EMBASE (Elsevier), MEDLINE (PubMed), Scopus (Elsevier), The Cochrane CENTRAL (Cochrane), and Web of Science (Clarivate), spanning from their inception to 17 November 2023. An updated search was conducted on 01/01/2025, using MEDLINE (PubMed); however, no new reviews have been published since the last search. Additionally, grey literature was explored through the ProQuest Dissertations & Theses Global database. Lastly, the reference lists of relevant studies were also searched.

### Search strategy

The search strategy, guided by the Peer Review of Electronic Search Strategies checklist^[^34^]^, incorporated both free-text and controlled vocabulary terms. Relevant search terms for “gastrointestinal surgery” and “chewing gum” were combined using Boolean operators with appropriate syntax (Supplementary Digital Content, Table A3, http://links.lww.com/JS9/D994). EndNote 21.1^[^35^]^ reference management software was used to handle article titles and abstracts retrieved from multiple databases, and duplicate records were *identified and removed.*

### Study selection

Two independent reviewers (SLL and SYY) assessed article titles and abstracts for eligibility, with full texts reviewed for articles meeting the criteria. Discrepancies were resolved after discussing with third reviewer (LJC). Relevant articles underwent data extraction, and inter-rater reliability (IRR) were assessed using Cohen’s kappa statistic (κ)^[^36^]^.

### Data extraction

SLL and SYY independently conducted data extraction from selected articles using data extraction tables, which includes author(s), publication year, objectives, sample size, delivery model (chewing gum), comparator, outcomes, review typology, number of included studies, geographical location, participants characteristics, search strategy and quality appraisal instruments. Review authors were contacted for clarification of information.

### Methodological quality assessment

This review methodology quality was assessed using the 16-item AMSTAR-2 (Assessment of Multiple Systematic Reviews 2) tool, featuring seven critical and nine non-critical domains^[^33^]^. Critical domains include pre-registering of protocol, thorough literature search, justifying excluded studies, assessing for risk-of-bias, appropriate meta-analytic methods, considering risk-of-bias during results interpreting and assessing the impact of publication bias. Two independent reviewers (SLL and SYY) assigned a quality rating (critically low, low, moderate, or high) to each systematic review based on these criteria^[^33^]^, and third reviewer (LJC) was consulted for any disagreement.

### Data synthesis

Random-effects meta-analyses were conducted using Jamovi software version 2.3.28^[^37^]^ to analyze both review-level and study-level data, preventing overestimating treatment effects by accounting for overlapping studies across different reviews^[^38^]^. For review-level data, effect size estimates were aggregated to mean differences using generic inverse variance methods, thereby pooling overall effect size estimates^[^39^]^. For study-level data, effect size was aggregated from the mean and standard deviation of non-overlapping primary studies^[^39^]^. Cochran’s Q (*X*^2^) and Higgins and Thompson’s *I*^2^ statistics assessed the heterogeneities between-review and between-study, with 50% indicating heterogeneity^[^39^]^. Data were grouped into surgical approach, patient cancer status, chewing frequency and duration for subgroup analyses to compare effect sizes^[^40^]^. Publication bias was assessed by examining asymmetry through funnel plot analysis^[^41^]^ and Egger’s test was conducted to detect small-study effect at a statistical significance level of *P* < 0.05^[^42^]^.

### Assessing certainty of evidence

To assess meta-analyses credibility^[^43^]^, the following criteria were applied (1: *P* < 10^−6^), (2: >1000 participants), (3: low or moderate heterogeneity, *I*^2^ < 50%), (4: 95% prediction interval (PI) excluding the null value), (5: no small-study effects) and (6: no excess significance bias). Evidence is categorized into five levels: (Class I, convincing: met all six criteria), (Class II, highly suggestive: met criteria 1–4), (Class III, suggestive: met criteria 2 and had *P* < 0.001), (Class IV, weak: *P* < 0.05) and (Class V, not significant: *P* ≥ 0.05).

### Overlapping studies within and between reviews

Measurement of study overlaps was assessed using percentage overlaps, covered area (CA) and validated corrected cover area (CCA)^[^44^]^ using these formulas:

$$\%Overlaps=\frac{Numberofoverlappedprimarypublications}{Numberofprimarypublications},CA=\frac{N}{rc},CCA=\frac{N-r}{rc-r}$$

*N*: sum of primary publications in the reviews, *r*: number of primary publications and c: number of reviews. The CCA score classifies the extent of overlap into the following categories: (slight: 0–5%), (moderate: 6–10%), (high: 11–15%) or (extremely high: >15%)^[^44^]^.

## Result

### Review selection

**Figure 1.**
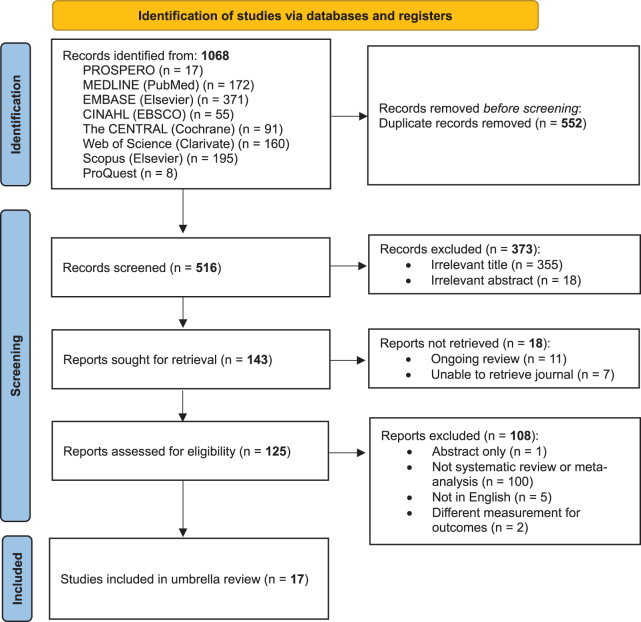
PRISMA flow diagram.

A comprehensive search across eight databases identified 1068 records. After removing 553 duplicates, 516 titles and abstracts were screened, leading to 125 full-text eligibility assessments. Ultimately, 17 reviews were included^[^3-8,18-28^]^, with exclusions documented in Table A4. Results were summarized using the PRISMA flow diagram (Fig. 1). Inter-rater reliability (IRR) was high, with kappa values for review selection (κ = 0.82), data extraction (κ = 0.88), AMSTAR-2 (κ = 0.81), and meta-analyses credibility (κ = 0.81).

### Review characteristics

Seventeen systematic reviews, involving 26 672 patients across 286 primary studies, are summarized in Tables 1-2. These include eight systematic reviews with meta-analyses, two standalone reviews, and seven meta-analyses. Database searches ranged from two^[^3^]^ to seven^[^19,20^]^, and the reviews covered publications from 2007^[^8^]^ to 2023^[^18^]^. Primary studies spanned 2002–2022, with sample sizes from 158^[^8,27,28^]^ to 9072 patients^[^19^]^. Four reviews provided geographical information on the conducted trials, encompassing regions such as North America, Asia, Europe, Africa, and Oceania^[^4,19,21,23^]^. Sinz, Warschkow^[^18^]^ compared chewing gum with coffee/caffeine, while 16 others examined chewing gum versus standard postoperative care. Table 3 presents an overview of the outcomes following chewing gum intervention from each review.

**Table 1:** Summary of systematic reviews

Authors, Year	Objectives	Sample size	Delivery	Comparator	Outcomes	AMSTAR
Sinz *et al* (2023)	To evaluate how coffee consumption, caffeine intake, and chewing gum impact the timing of initial passing of gas, the timing of the first bowel movement, and the duration of hospital stay in postoperative patients.	4999	Chewing gum	Coffee Caffeine	1, 2, 3	Low
Roslan *et al* (2020)	To provide a valid and up-to-date summary of relevant high-quality trials comparing the impact of chewing gum compared to standard care (the use of controls or placebos) in the management of POI in adults undergoing resectional large bowel surgery with or without anastomosis.	970	Chewing gum	Standard postoperative care	1, 2, 3, 4, 5	Low
Liu *et al* (2017)	To further evaluate the effect of chewing gum on ameliorating ileus following colorectal surgery.	1736	Chewing gum	Standard postoperative care	1, 2, 3, 6	Critically low
Mei *et al* (2017)	To review the current evidence on the influence of gum chewing on intestinal function and to reassess the efficacy of chewing gum in intestinal function recovery after colorectal surgery.	1845	Chewing gum	Standard postoperative care	1, 2, 3, 4, 5, 7, 8, 9, 10, 11	Critically low
Song *et al* (2016)	To evaluate the effect and safety of chewing gum versus standard postoperative care protocol after colorectal surgery.	2214	Chewing gum	Standard postoperative care	1, 2, 3, 4, 5, 7, 8, 9, 10, 12, 13, 14, 15, 16, 17, 18, 19, 20, 21, 22	Critically low
Short *et al* (2015)	To examine whether chewing gum after surgery hastens the return of gastrointestinal function.	9072	Chewing gum	Standard postoperative care	1, 2, 3, 5, 12, 13, 14, 21, 23, 24	High
Su’a *et al* (2015)	To evaluate the efficacy and safety of chewing gum in treating POI.	1019	Chewing gum	Standard postoperative care	1, 2, 3, 14	Critically low
Ho *et al* (2014)	To reassess the effect of sham feeding on the return of gastrointestinal tract function, length of stay, and postoperative morbidity following colorectal surgery.	612	Chewing gum	Standard postoperative care	1, 2, 3, 13, 21, 22	Low
Li *et al* (2013)	To accurately assess whether the use of chewing gum could reduce the duration of postoperative ileus following abdominal surgery.	1374	Chewing gum	Standard postoperative care	1, 2, 3, 13	Critically low
Yin *et al* (2013)	To study the effects of gum chewing in the postoperative period after abdominal surgery.	1148	Chewing gum	Standard postoperative care	1, 2, 3	Critically low
Fitzgerald & Ahmed (2009)	To identify clinical trials of chewing-gum therapy in relation to postoperative ileus and analyze results by meta-analysis to show what benefit.	272	Chewing gum	Standard postoperative care	1, 2, 3, 13	Critically low
Nobel *et al* (2009)	To assess the evidence for benefit and harm from chewing gum following elective intestinal surgery.	437	Chewing gum	Standard postoperative care	1, 2, 3	Critically low
Parnaby *et al* (2009)	To assess current evidence for gum chewing and gut function.	256	Chewing gum	Standard postoperative care	1, 2, 3, 14	Critically low
Vásquez *et al* (2009)	To investigate the effects of gum chewing on ileus after elective colonic surgery.	244	Chewing gum	Standard postoperative care	1, 2, 3	Critically low
de Castro *et al* (2008)	To analyze whether gum chewing facilitates recovery from postoperative ileus in patients undergoing colorectal surgery.	158	Chewing gum	Standard postoperative care	1, 2, 3	Critically low
Purkayastha *et al* (2008)	To compare outcomes following abdominal surgery with or without the use of chewing gum in the early postoperative period.	158	Chewing gum	Standard postoperative care	1, 2, 3	Critically low
Chan & Law (2007)	To conduct a systematic review of all relevant trials on chewing gum to reduce postoperative ileus after colorectal resection.	158	Chewing gum	Standard postoperative care	1, 2, 3, 13, 21, 22	Critically low

1 = Time to first flatus, 2 = Time to first bowel movement, 3 = Length of stay, 4 = Postoperative ileus, 5 = Mortality, 6 = Clinically relevant parameters, 7 = Time to first feeding, 8 = Postoperative nausea, 9 = Postoperative vomiting, 10 = Postoperative abdominal distention, 11 = Postoperative pneumonia, 12 = Time to first bowel sound, 13 = Overall complication rate, 14 = Other complications, 15 = Wound infection, 16 = Other infections, 17 = Bleeding, 18 = Wound dehiscence, 19 = Anastomotic leak, 20 = Complications related to chewing gum, 21 = Readmission rate, 22 = Reoperation rate, 23 = Tolerability of gum, 24 = Costs and benefits

**Table 2:** Characteristics of included reviews

Authors, Year	Review typology	Number of included studies (type)	Geographical location	Participant characteristics		Search strategy				Quality appraisal instruments
				Gender	Mean age range	Number of databases	Search Periods	Publication range of year	Publication language	
Sinz *et al* (2023)	SR, MA	32 (RCT)	NR	Male	NR	3	Inception to 8th Aug 2022	2006–2022	No restriction	ROB-2 tool.
				Female						
Roslan *et al* (2020)	SR, MA	16 (RCT)	NR	Male	>16	2	Between 2000 and 2019	2002–2018	English	Jadad scale
				Female						Cochrane Risk of Bias Tool
Liu *et al* (2017)	MA	18 (RCT)	North America (7)	Male	>18	3	Inception to Feb 2018	2002–2017	NR	Cochrane Risk of Bias Tool
				Female						
			Asia (6)							
			Europe (4)							
			Oceania (1)							
Mei *et al* (2017)	SR, MA	17 (RCT)	NR	Male	NR	4	Inception to Apr 2017	2002–2017	English	Cochrane Risk of Bias Tool
				Female						
Song *et al* (2016)	MA	26 (RCT)	NR	Male	42.1–70.6	5	Inception to 31 January 2016 (Updated 31 May 2016)	2002–2016	No restriction	Cochrane Risk of Bias Tool
				Female						
Short *et al* (2015)	SR, MA	81 (RCT)	Asia (54)	Male	5–70.62	7	Inception to Jun 2013	2002–2014	No restriction	Cochrane Risk of Bias Tool
			North America (12)	Female						
			Europe (3)				(Updated Jun 2014)			
Su’a *et al* (2015)	SR, MA	12 (RCT)	NR	Male	NR	7	Feb to Apr 2013	2002–2013	English	Jadad score
				Female						Cochrane Risk of Bias Tool
Ho *et al* (2014)	MA	10 (RCT)	NR	Male	>16	5	Inception to Apr 2013	2002–2013	No restriction	Cochrane Risk of Bias Tool
				Female						
Li *et al* (2013)	MA	17 (RCT)	Asia (7)	Male	7–68	4	Inception to Dec 2012	2002–2012	No restriction	Cochrane Risk of Bias Tool
			North America (5)							
				Female						
			Europe (3)							
			Africa (2)							
Yin *et al* (2013)	MA	14 (RCT)	Asia (8)	Male	7-68	3	Inception to Feb 2012	2002–2011	No restriction	Cochrane Risk of Bias Tool
			North America (3)							
			Europe (2)							
			Africa (1)							

				Female						
Fitzgerald & Ahmed (2009)	SR, MA	7 (RCT)	NR	Male	NR	3	NR	2002–2006	No restriction	Jadad scale
				Female						
Nobel *et al* (2009)	SR, MA	9 (RCT)	NR	Male	7–71	3	NR	2002–2008	NR	NR
				Female						
Parnaby *et al* (2009)	MA	6 (RCT)	NR	Male	55.6–68	4	Inception to Jul 2008	2002–2008	No restriction	Jadad scale
				Female						
Vásquez *et al* (2009)	SR, MA	6 (RCT)	NR	Male	>15	4	Inception to Aug 2008	2002–2006	English	NR
				Female						
de Castro *et al* (2008)	SR	5 (RCT)	NR	Male	54–68	3	Inception to Jun 2007	2002–2006	English	NR
				Female						
Purkayastha *et al* (2008	MA	5 (RCT)	NR	Male	55.6–68	4	Inception to 18th Jul 2006	2002–2006	No restriction	Jaded scale
				Female						
Chan & Law (2007)	SR	5 (RCT)	NR	Male	61.9	6	Inception to Jan 2007	2002–2006	English	Jadad scale
				Female						

SR: Systematic Review; MA: Meta-analysis; RCT: Randomized Controlled Trial; NR: Not Reported.

**Table 3:** Overview of outcomes after chewing gum intervention from each of the reviews

References	Outcomes									
	Time to first flatus	Time to first bowel movement	Length of stay	Postoperative ileus	Nausea	Vomiting	Bloating	Readmission	Reoperation	Mortality
Sinz *et al* (2023)	▼	▼	▼							
Roslan *et al* (2020)	▼	▼	NS	▼						NS
Liu *et al* (2017)	▼	▼	▼	▼	NS	NS	NS	NS	NS	
Mei *et al* (2017)	▼	▼	▼	NS	NS	NS	NS			NS
Song *et al* (2016)	▼	▼	▼	▼	NS	NS	NS	NS	NS	NS
Short *et al* (2015)	▼	▼	▼							
Su’a *et al* (2015)	▼	▼	▼							
Ho *et al* (2014)	▼	▼	▼					NS	NS	
Li *et al* (2013)	▼	▼	▼							
Yin *et al* (2013)	▼	▼	▼							
Fitzgerald & Ahmed (2009)	▼	▼	NS							
Nobel *et al* (2009)	▼	▼	▼							
Parnaby *et al* (2009)	▼	▼	NS							
Vásquez *et al* (2009)	▼	▼	NS							
de Castro *et al* (2008)	▼	▼	NS							
Purkayastha *et al* (2008)	▼	▼	NS							
Chan & Law (2007)	▼	▼	▼					NS	NS	

▼ = Reduced, NS = Not Significant

### Methodological quality assessment of systematic reviews

Seventeen reviews underwent methodological quality assessment. According to AMSTAR-2 results (Supplementary Digital Content, Table A5, http://links.lww.com/JS9/D994), one review^[^19^]^ was rated high quality, three reviews^[^3,7,18^]^ as low quality, and the remaining thirteen as critically low quality. Key issues included lack of justification for exclusions (82.35%), protocol registration (76.47%), and appropriate statistical methods (41.18%). Risk of bias interpretation (35.29%) and publication bias investigation (23.53%) were also lacking. All reviews partially met criteria for comprehensive searches.

### Credibility of meta-analyses

Analysis was conducted on data from 46 distinct meta-analyses found in 15 systematic reviews. Supplementary Digital Content, Table A6 (http://links.lww.com/JS9/D994) summarizes the effect of chewing gum on TFF, TFBM and LOS. Four out of six criteria were lacking in most reviews (criteria 1, 3, 4, and 5). No reviews presented results reaching a significance level of *P* < 10^-6^, and none reported a 95% PI. Moderate-to-large heterogeneity (*I*^2^ > 50%) were seen in 93.48% of the meta-analyses. In terms of the credibility of the association between chewing gum and TFF, three out of 15 meta-analyses (20%) provided suggestive evidence, while the remaining 12 presented weak evidence. For the association with TFBM, two out of 16 meta-analyses (12.5%) offered suggestive evidence, 13 reported weak evidence, and one found non-significant evidence. Regarding LOS, two out of 15 meta-analyses (13.3%) provided suggestive evidence, eight reported weak evidence, and five revealed non-significant evidence.

### Overlapping of primary studies

The percentage of overlaps for between-studies across 17 reviews was 37.86%, CA was 0.16 and CCA was high (11.10%) (Supplementary Digital Content, Tables A7–8, http://links.lww.com/JS9/D994).

### Time to first flatus

Meta-analysis (Supplementary Digital Content, Figure A1.1, http://links.lww.com/JS9/D994) showed chewing gum reduced TFF by −0.36 days (95% CI: −0.61, −0.1; *P* = 0.007, 4 reviews) or −12.26 hours (95% CI: −14.73, −9.78; *P* < 0.001, 11 reviews). Evaluating the credibility of evidence, 20% (three out of 15) of the meta-analyses provided suggestive evidence (Class III), while the remaining 80% yielded weak evidence (Class IV). Study-level analysis (57 studies) confirmed reductions of −11.33 hours (95% CI: −13.88, −8.79; *P* < 0.001) (Table 4, Supplementary Digital Content, Figure A1.2, http://links.lww.com/JS9/D994).

**Table 4:** Meta-analysis of outcomes of chewing gum intervention on meta-analyzed data and study-level data of TFF, TFBM and LOS

Outcome	Meta-analyzed data							Study-level data						
		MA	Effect size (MD) (95% CI)	*P*-value	*I*^2^ (%)	Egger’s test (*P*-value)		Classification	Subgroups	No. of trials (*N*)	Effect size (MD) (95% CI)	*P*-value	*I*^2^ (%)	Subgroup differences, *P*-value
TFF	Days	4	−0.36 (–0.61, −0.1)	0.007	5.27%			Global analysis	–	57 (5909)	−11.33 (−13.88, −8.79)	<0.001	97.74%	–
	Hours	11	−12.26 (–14.73, −9.78)	<0.001	89.05%	<0 .001		Surgical approach	Open surgery	25 (1368)	−13.74 (−17.95, −9.52)	<0 .001	91.63%	−2.57 *P* = 0.054
									Laparoscopic surgery	8 (802)	−9.02 (−14.86, −3.18)	0.002	98.19%	
									Mix	15 (2483)	−6.58 (−11.41, −1.74)	0.008	96.22%	
								Cancer condition	Cancer	18 (1792)	−11.72 (−16.97, −6.48)	<0 .001	97.73%	0.33 *P* = 0.734
									Non-cancer	6 (402)	−15.17 (−20.89, −9.46)	<0 .001	90.59%	
									Mix	9 (1215)	−6.4 (−13.62, 0.83)	0.083	56.47%	
								Chewing gum frequency	≤2x/days	4 (843)	−13.98 (−24.21, −3.75)	0.007	99.58%	1.68 *P* = 0.439
									3x/days	41 (3812)	−11.63 (−14.76, −8.51)	<0.001	96.62%	
									>4x/days	11 (1311)	−8.76 (−14.12, −3.4)	0.001	96.61%	
								Chewing gum duration	<30 min	32 (3715)	−11.13 (−14.3, −7.96)	<0.001	98.38%	−1.05 *P* = 0.539
									≥30 min	17 (1770)	−10.77 (−16.4, −5.15)	<0 .001	96.16%	
									≥45 min	6 (371)	−11.31 (−18.62, −4)	0.002	40.61%	
TFBM	Days	5	−0.59 (−0.94, −0.23)	0.001	8.87%			Global analysis	–	49 (5278)	−16.07 (−20.23, −11.92)	<0 .001	98.95%	–
	Hours	11	−19.29 (−23.79, −14.79)	<0 .001	95.57%	<0.001		Surgical approach	Open surgery	23 (1267)	−18.52 (−24.6, −12.45)	<0 .001	94.32%	1.53 *P* = 0.407
									Laparoscopic surgery	6 (613)	−16.5 (−30.61, −2.39)	0.022	99.61%	
									Mix	14 (2470)	−11.96 (−19.6, −4.33)	0.002	96.13%	
								Cancer condition	Cancer	15 (1589)	−18.36 (−26.39, −10.34)	<0 .001	98.57%	1.6 *P* = 0.327
									Non-cancer	6 (402)	−20.83 (−25.15, −16.51)	<0 .001	69.45%	
									Mix	8 (1217)	−9.61 (−24.71, 5.48)	0.212	89.13%	
								Chewing gum frequency	≤2x/days	3 (733)	−18.35 (−40.77, 4.08)	0.109	99.90%	2.84 *P* = 0.419
									3x/days	35 (3223)	−16.92 (−22.56, −11.27)	<0 .001	98.35%	
									>4x/days	10 (1249)	−14.19 (−17.65, −10.72)	<0 .001	88.65%	
								Chewing gum duration	<30 min	27 (3268)	−15.69 (−20.47, −10.91)	<0 .001	98.81%	−1.97 *P* = 0.461
									≥30 min	14 (1592)	−14.88 (−22.87, −6.89)	<0 .001	98.73%	
									≥45 min	6 (365)	−13.95 (−29.28, 1.38)	0.075	79.95%	
LOS	Days	12	−0.85 (−1.22, −0.48)	<0 .001	45.09%	0.12		Global analysis	–	44 (5591)	−0.98 (−1.31, −0.65)	<0 .001	89.10%	–
	Hours	3	−20.08 (−28.62, −11.54)	<0 .001	82.17%			Surgical approach	Open surgery	23 (1339)	−1.13 (−1.58, −0.69)	<0 .001	90.16%	0.06 *P* = 0.698
									Laparoscopic surgery	5 (383)	−0.61 (−1.76, 0.53)	0.294	83.65%	
									Mix	12 (3491)	−0.59 (−1.16, −0.02)	0.042	73.93%	
								Cancer condition	Cancer	12 (1448)	−0.81 (−1.66, 0.05)	0.065	81.58%	−0.09 *P* = 0.521
									Non-cancer	7 (450)	−0.96 (−1.35, −0.57)	<0 .001	78.36%	
									Mix	7 (2323)	−0.98 (−1.82, −0.13)	0.024	67.14%	
								Chewing gum frequency	3x/days	31 (4048)	−0.92 (−1.29, −0.54)	<0 .001	84.23%	0.18 *P* = 0.532
									>4x/days	9 (1139)	−0.79 (−1.31, −0.28)	0.003	76.32%	
								Chewing gum duration	<30 min	24 (2624)	−1.02 (−1.5, −0.53)	<0 .001	87.61%	−0.12 *P* = 0.555
									≥30 min	12 (2480)	−0.8 (−1.19, −0.4)	<0 .001	77.26%	
									≥45 min	5 (322)	−0.59 (−1.33, 0.15)	0.116	48.18%	


CI = Confidence interval; MA = Number of meta-analyses; No = Number; TFF = Time to first flatus; TFBM = Time to first bowel movement; LOS = Length of stay.

Subgroup analyses indicate a reduction in TFF, though subgroup differences were not statistically significant (*X*^2^ = −2.57–1.68, *P* = 0.054–0.734). Subgroups indicated greater reductions for open surgery (−13.7 hours, 95% CI = −17.95, −9.52), non-cancer patients (−15.17 hours, 95% CI = −20.89, −9.46), and chewing ≥45 minutes (−11.31 hours, 95% CI = −16.97, −6.48), with consistent reductions across chewing frequencies. Considerable heterogeneity was observed across all subgroups; however, no statistically significant differences were observed among them (Supplementary Digital Content, Figures A1.3–6, http://links.lww.com/JS9/D994).

### Time to first bowel movement

Meta-analysis (Supplementary Digital Content, Figure A2.1, http://links.lww.com/JS9/D994) showed chewing gum reduced TFBM by −0.59 days (95% CI: −0.94, −0.23; *P* = 0.001, 5 reviews) or −19.29 hours (95% CI: −23.79, −14.79; *P* < 0.001, 11 reviews). The credibility evidence showed that 12.5% (two out of 16 of the meta-analyses) provided suggestive evidence (Class III), while the remaining 81.25% as weak evidence (Class IV), with one rated as non-significant (Class V). Study-level analysis (49 studies) confirmed reductions of −16.07 hours (95% CI: −20.23, −11.92; *P* < 0.001) (Table 4, Supplementary Digital Content, Figure A2.2, http://links.lww.com/JS9/D994).

Subgroup analyses indicate a reduction in TFBM, though subgroup differences were not statistically significant (*X*^2^ = −1.97–2.84, *P* = 0.327–0.461). Reductions were greater for open surgery (−18.52 hours, 95% CI = −24.6, −12.45) and non-cancer patients (−20.83 hours, 95% CI = −25.15, −16.51). Chewing 3x/day (−16.92 hours, 95% CI = −22.56, −11.27) and < 30 minutes (−15.69 hours, 95% CI = −20.47, −10.91) were most effective. Substantial heterogeneity was observed across all subgroups; however, no statistically significant differences were observed among them (Figures A2.3–6, Supplementary Digital Content, http://links.lww.com/JS9/D994).

### Length of stay

Meta-analysis (Figure A3.1, Supplementary Digital Content, http://links.lww.com/JS9/D994) showed chewing gum reduced LOS by −0.85 days (95% CI: −1.22, −0.48; *P* < 0.001, 12 reviews) or −20.08 hours (95% CI: −28.62, −11.54; *P* < 0.001, 3 reviews). The credibility evidence showed that 13.33% (two out of 16 of the meta-analyses) provided suggestive evidence (Class III), while 53.33% as weak evidence (Class IV), and 33.33% as non-significant (Class V). Study-level analysis (44 studies) confirmed reductions of −0.98 days (95% CI: −1.31, −0.65; *P* < 0.001) (Table 4, Figure A3.2, Supplementary Digital Content, http://links.lww.com/JS9/D994).

Subgroup analyses indicate a reduction in LOS, though subgroup differences were not significant (*X*^2^ = −0.09–0.18, *P* = 0.521–0.698). Reductions were greater for open surgery (−1.13 days, 95% CI = −1.58, −0.69) and non-cancer patients (−0.96 days, 95% CI = −1.35, −0.57). Chewing 3x/day (−0.92 days, 95% CI = −1.29, −0.5) and < 30 minutes (−1.02 days, 95% CI = −1.5, −0.53) showed the most benefit. Despite considerable heterogeneity, no statistically significant differences were found among the subgroups (Figures A3.3–6, Supplementary Digital Content, http://links.lww.com/JS9/D994).

### Complications

**Figure 2.**
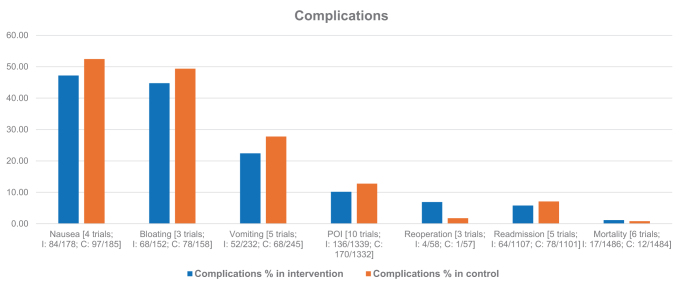
Bar chart of study-level data for complications in intervention and control groups. *Note*. I = Intervention group; C = Control group; POI = Postoperative ileus.

Figure 2 summarizes complications. The intervention group had lower rates of nausea (47.19% vs. 52.43%), bloating (44.74% vs. 49.37%), vomiting (22.41% vs. 27.76%), postoperative ileus (10.16% vs. 12.76%), and readmission (5.78% vs. 7.08%). Higher rates were seen for reoperation (6.90% vs. 1.75%) and mortality (1.14% vs. 0.81%).

## Discussion

This umbrella review synthesizes evidence on the efficacy of chewing gum in improving postoperative recovery, particularly focusing on TFF, TFBM, LOS, and postoperative complications. Drawing upon data from eight systematic reviews with meta-analyses, two systematic reviews, and seven meta-analyses, this review encompasses a significant sample of 26 672 patients from 286 primary studies. Despite the preponderance of weak evidence (71.74%) and a smaller proportion of non-significant (13.04%) and suggestive findings (15.22%), the aggregated data suggest that chewing gum may lead to a substantial reduction in TFF, TFBM, and LOS.

The consistent findings across the included reviews demonstrate that chewing gum significantly accelerates the return of gastrointestinal function, as evidenced by reductions in TFF and TFBM^[^29^]^. These outcomes are biologically plausible given the physiological responses triggered by chewing gum. Chewing stimulates salivary production and increases gastric and pancreatic secretions, which collectively enhance gastrointestinal motility^[^17^]^. Furthermore, sorbitol, a common ingredient in many chewing gums, acts as an osmotic laxative, drawing water into the colon and thereby promoting bowel movements^[^45,46^]^. This dual mechanism – both mechanical and chemical – offers a compelling explanation for the observed benefits in postoperative recovery. However, it is important to consider other factors that may influence recovery, such as the patient’s nutritional status. Studies have shown that patients arriving in good nutritional condition experience better recovery and fewer complications^[^47^]^, highlighting the need to address nutritional optimization alongside interventions like chewing gum. The impact of chewing gum on TFF and TFBM is particularly noteworthy given the implications for patient care. Delayed return of bowel function is a common postoperative complication that can lead to extended hospital stays and increased healthcare costs. By significantly reducing TFF and TFBM, chewing gum presents a simple, non-invasive intervention that can be easily incorporated into postoperative care routines. The consistency of these findings across a large and diverse patient population further underscores the potential of chewing gum to improve postoperative outcomes broadly.

Reduction in LOS is a critical outcome in postoperative care, with direct implications for healthcare resource utilization and patient throughout. Our review found that chewing gum significantly reduces LOS, a finding consistent across multiple studies^[^18^]^. This reduction is not only beneficial for patients – by shortening their recovery time and reducing their exposure to hospital-related complications^[^48^]^ – but also advantageous for healthcare systems, which can achieve more efficient use of resources and lower overall costs. Given the simplicity and low cost of this intervention, its integration into clinical practice could be both cost-effective and beneficial on a large scale. Moreover, the reduction in LOS may contribute to a quicker turnover of hospital beds, which could help ease pressures on healthcare facilities, especially in high-demand settings.

Interestingly, the subgroup analyses conducted within this review did not reveal substantial differences in the efficacy of chewing gum across various patient groups, surgical types, or intervention protocols for TFF, TFBM, and LOS. This lack of significant variation suggests that the benefits of chewing gum are broadly applicable and not heavily dependent on specific patient characteristics or surgical contexts. The uniformity in results may reflect the fundamental physiological mechanisms by which chewing gum aids recovery, such as increased salivation and gastric secretions, which appear to operate consistently across different scenarios. The consistent effect of chewing gum on LOS observed across various patient demographics and surgical types may suggest that it could be widely applicable as a standard part of postoperative care protocols^[^29^]^. However, it is important to consider the potential influence of surgical approach on postoperative bowel recovery, particularly the differences between open and minimally invasive techniques. Open surgery is associated with greater peritoneal trauma, fluid shifts, and bowel handling, all of which contribute to prolonged postoperative ileus^[^49^]^. In contrast, laparoscopic and robotic-assisted techniques are less invasive, involve minimal peritoneal cavity disruption, and reduce bowel manipulation, leading to faster gastrointestinal recovery^[^49^]^. Some studies have reported shorter time to first flatus and bowel movement following minimally invasive procedures compared to open surgery, suggesting that the baseline recovery trajectory differs between these approaches^[^49^]^. While trends in our review indicate benefits of chewing gum in both surgical techniques, the smaller number of studies specifically examining robotic or laparoscopic procedures may have limited the statistical power to detect nuanced differences in efficacy^[^50,51^]^. For example, while trends suggest benefits in both open and laparoscopic surgeries, and among cancer and non-cancer patients, the smaller number of studies in some subgroups could obscure finer variations in efficacy. This limitation underscores the need for larger, more focused studies to explore potential subgroup-specific effects more comprehensively.

Our analysis also highlights the potential of chewing gum to reduce postoperative complications such as nausea, bloating, vomiting, and POI compared to the control group. Although some of the included reviews reported non-significant findings^[^4-6^]^, the overall trend indicates a beneficial effect. Besides postoperative complications, four reviews^[^4,6-8^]^ assessed readmission and reoperation rates. While the intervention group experienced fewer readmissions, there was no significant reduction in reoperations, likely due to the small number of studies included. Furthermore, three reviews^[^3,5,6^]^ evaluated mortality rates and found no significant decrease with chewing gum compared to the control group. Importantly, none of the studies reviewed identified chewing gum as a direct cause of any adverse events, further supporting its safety profile. This aspect is particularly relevant given the growing emphasis on enhancing patient safety and minimizing complications in postoperative care. The ability of chewing gum to reduce common and distressing postoperative symptoms could contribute significantly to patient comfort and satisfaction, as well as to the overall quality of care.

### Implications to clinical practice and research

The findings of this review suggest that chewing gum could be a valuable adjunct to standard postoperative care, offering a simple and cost-effective means of enhancing patient recovery. Its ability to reduce TFF, TFBM, LOS, and complications positions it as a potentially impactful intervention that could be easily adopted in a wide range of surgical contexts. However, the overall quality of the evidence remains a significant concern. Many of the included reviews and meta-analyses were of low methodological quality, which limits the conclusiveness of the findings. Given its demonstrated ability to enhance patient outcomes and safety, chewing gum emerges as a promising measure within postoperative care protocols. Policymakers and clinical leaders could consider its inclusion in postoperative care strategies to empower clinical staff and improve patient recovery and healthcare efficiency.

To enhance methodological rigor, future systematic reviews should include a comprehensive list of excluded articles with reasons, preregister their protocols, employ appropriate statistical methodologies, consider individual study biases, investigate publication bias, and use robust techniques to assess the risk of bias. Many meta-analyses in the selected reviews used sample sizes below 1000, highlighting the need for larger clinical trials for more definitive evidence. Six reviews^[^3-8^]^ attempted to meta-analyze the effects of chewing gum on postoperative complications but yielded inconclusive results due to limited trials. Further well-designed clinical trials are needed to investigate the effects of chewing gum on postoperative complications, enhancing evidence robustness and yielding conclusive results. Additional trials could allow future reviews to explore factors contributing to variability in postoperative complications, including patient populations, surgical procedures, and chewing gum protocols.

### Strengths and limitations

This umbrella review has several strengths, including a comprehensive assessment of the credibility of the included meta-analyses^[^43^]^ and a thorough evaluation of study overlap^[^44^]^, which helps mitigate the risk of overestimating treatment effects due to redundant data. However, several limitations must be acknowledged. The high heterogeneity observed across the included studies may have affected the accuracy of the pooled estimates, and the generalizability of the findings is limited by the inclusion of reviews published only in English. Furthermore, the low quality of many of the included reviews and the weak evidence provided by over half of the meta-analyses necessitate caution in interpreting the results.

Another key limitation is the inability to account for distinctions between upper and lower gastrointestinal surgeries, as well as resectional versus non-resectional procedures, due to inconsistent reporting, limiting our ability to assess their impact on outcomes. Additionally, the potential effects of prior abdominal surgeries, particularly the presence of adhesions, were not adequately addressed in the included reviews. While these factors are recognized as important effect modifiers, the lack of detailed reporting in the included studies restricted our ability to conduct a comprehensive subgroup analysis. Future research should aim to provide more granular data to better account for these variations.

## Conclusion

This umbrella review found that chewing gum may result in a large reduction in TFF, TFBM, LOS, and postoperative complications. Implementing chewing gum as an adjunct to standard postoperative care can enhance gastrointestinal recovery after surgery. While subgroup analyses did not identify significant moderators, better effects were observed in open surgery, non-cancer populations, chewing no more than twice per day, and chewing for less than 30 minutes. However, due to the low quality of included reviews and limited evidence, additional well-designed reviews are needed.

## Data Availability

Data and materials are available to strengthen the transparency and reliability of the study. Additional data are available upon request by sending an email to the corresponding author.

## Research registration unique identifying number (UIN)

PROSPERO Number: CRD42024519952.
